# Predators in the Dark: Metabarcoding Reveals Arcellinida Communities Associated with Bat Guano, Endemic to Dinaric Karst in Croatia

**DOI:** 10.1007/s00248-024-02483-z

**Published:** 2025-01-06

**Authors:** Ángel García-Bodelón, Najla Baković, Emilio Cano, Fernando Useros, Enrique Lara, Rubén González-Miguéns

**Affiliations:** 1 Real Jardín Botánico (RJB-CSIC), C/ Moyano 1, 28014 Madrid, Spain; 2 Universidad Complutense de Madrid, 28040 Madrid, Spain; 3 Laboratory for Flora, Fauna and Habitats, DVOKUT-ECRO Ltd., Zagreb, Croatia; 4 ADIPA – Society for Research and Conservation of Croatian Natural Diversity, Zagreb, Croatia; 5 Research Support Unit, Real Jardín Botánico (CSIC), C/ Moyano 1, 28014 Madrid, Spain; 6 Institut de Biologia Evolutiva (CSIC-Universitat Pompeu Fabra), 08003 Barcelona, Spain

**Keywords:** Arcellinida, Karst caves, Biodiversity, Bat guano, Metabarcoding

## Abstract

Karst caves, formed from the dissolution of soluble rocks, are characterized by the absence of photosynthetic activity and low levels of organic matter. Organisms evolve under these particular conditions, which causes high levels of endemic biodiversity in both macroorganism and microbes. Recent research has highlighted the presence of testate amoebae (Arcellinida) group in cave environments. This study investigates the diversity of Arcellinida in Dinaric karstic caves in Croatia, a global diversity hotspot, focusing on the influence of bat guano on community structure. Sediment samples were collected from two independent hydrosystems, and a metabarcoding approach was used to assess Arcellinida diversity at specific and intraspecific levels, using Arcellinid-specific primers to amplify the mitochondrial cytochrome oxidase subunit I (COI) region. Results reveal a significant impact of guano on both specific and intraspecific diversity of Arcellinida. Communities in guano-rich sites displayed higher diversity, abundance, and the presence of unique OTUs and genetic variants not observed in other habitats, highlighting the crucial role of bats as ecosystem engineers. In contrast, sites without guano hosted communities with low abundance and reduced biodiversity. These differences suggest the existence of guano-associated Arcellinida communities. This study provides new insights into the biodiversity of subterranean ecosystems and the ecological roles of Arcellinida in karstic environments.

**Supplementary Information:**

The online version contains supplementary material available at 10.1007/s00248-024-02483-z.

## Introduction

Cave environments in the world support astonishing endemic biodiversity that have long been in a focus of scientific inquiry. Spatial isolation, together with absence of light, stable microclimates and food scarcity played important roles in cave fauna evolution [1]. The absence of photoautotrophic organisms such as plants, algae and cyanobacteria prevents primary production (unless steep chemoclines are present) so that caves are naturally organic matter scarce environments. Although caves are often considered as isolated environments, they are constantly connected with the surface through water and air. Rain water is constantly seeping into the cave through soil and epikarst layer [2], and some caves are fed by exogenous (sinking) streams or endogenous streams (formed underground) [3]. These freshwater affluents are richer in organic matter [4], allowing the maintenance of the endemic cave biodiversity; but the most concentrated organic matter input in caves is provided by bat colonies through guano deposition [5]. Guano provides high amounts of nutrients for a variety of organisms in caves [6, 7]. It plays a crucial role as the trophic link connecting metazoans and microorganisms [8] and is therefore indispensable for maintaining a most of the diversity in these otherwise nutrient-depleted environments. While studies on bird-modified soils, such as those in Arctic ecosystems, have shown shifts in testate amoeba assemblages due to nutrient enrichment [9], the impact of guano-derived nutrients in cave environments remains largely unexplored. This gap highlights the importance of studying how bat guano affects Arcellinida communities in subterranean ecosystems.

Because of these characteristics, organisms living in caves have gone through strong adaptive selection and became strongly associated these environments, to the point that they cannot live elsewhere. There are several examples in metazoan organisms, like invertebrates (e.g. crustaceans, insects, spiders, bivalves, snails, oligochaetes) and in a few cases some vertebrates (e.g. cave olm) [10, 11]. All these organisms adapted their life history traits, morphology, physiology and behaviour to their environment [12]; they build small populations and have high endemism rates, which makes them especially susceptible to extinction [13].

Caves environments are also biodiversity hotspots for microorganisms such as fungi, protists, bacteria and archaea [14]. Among many cave protists [15–18], testate amoebae received special attention as their shells make them relatively easier to identify [19–23]. Rather intriguing was recent discovery of several new species for science within order Arcellinida (Tubulinea; Amoebozoa): *Centropyxis bipilata* (found exclusively in caves) [24], or *Difflugia* (now *Cylindrifflugia*) *alhadiqa* and *Heleopera baetica* [25]. Arcellinida are particularly well-known for their narrow ecological niches and high levels of geographical endemism [26, 27]. These characteristics make them a perfectly suited model group to study microbial evolution in caves. Moreover, the stable year-long environmental conditions within the cave favour K-selected organisms [1] such as Arcellinida. But on the other hand, these organisms are microbial top-predators, and their presence depends on the presence of preys, which consist in smaller protists, metazoa, fungi, and organic debris [28]. For these reason, the presence of Arcellinida stable populations depends on organic inputs, which can be potentially provided by bat guano.

The main objective of this study is to compare microbial diversity between cave and surface environments, focusing on Arcellinida communities in Dinaric karstic caves. Additionally, we will determine the role played by bat guano microenvironments in their diversity structuring; in other terms, is there a “guanophilic” community in Arcellinida? In order to answer these questions, we followed two independent streams in the Dinaric karst (Croatia) and sampled sediment before, during (subterranean) and after their passage within a cave and compared these data with communities present in cave pool with guano. In order to evaluate the diversity of Arcellinida, we applied a specific metabarcoding approach [29], which allows diversity assessments at the species and intraspecific level. Our results will partially fill the gap on protists connectivity between surface and subsurface cave habitats.

## Material and Methods

### Study Area and Sampling

**Fig. 1:**
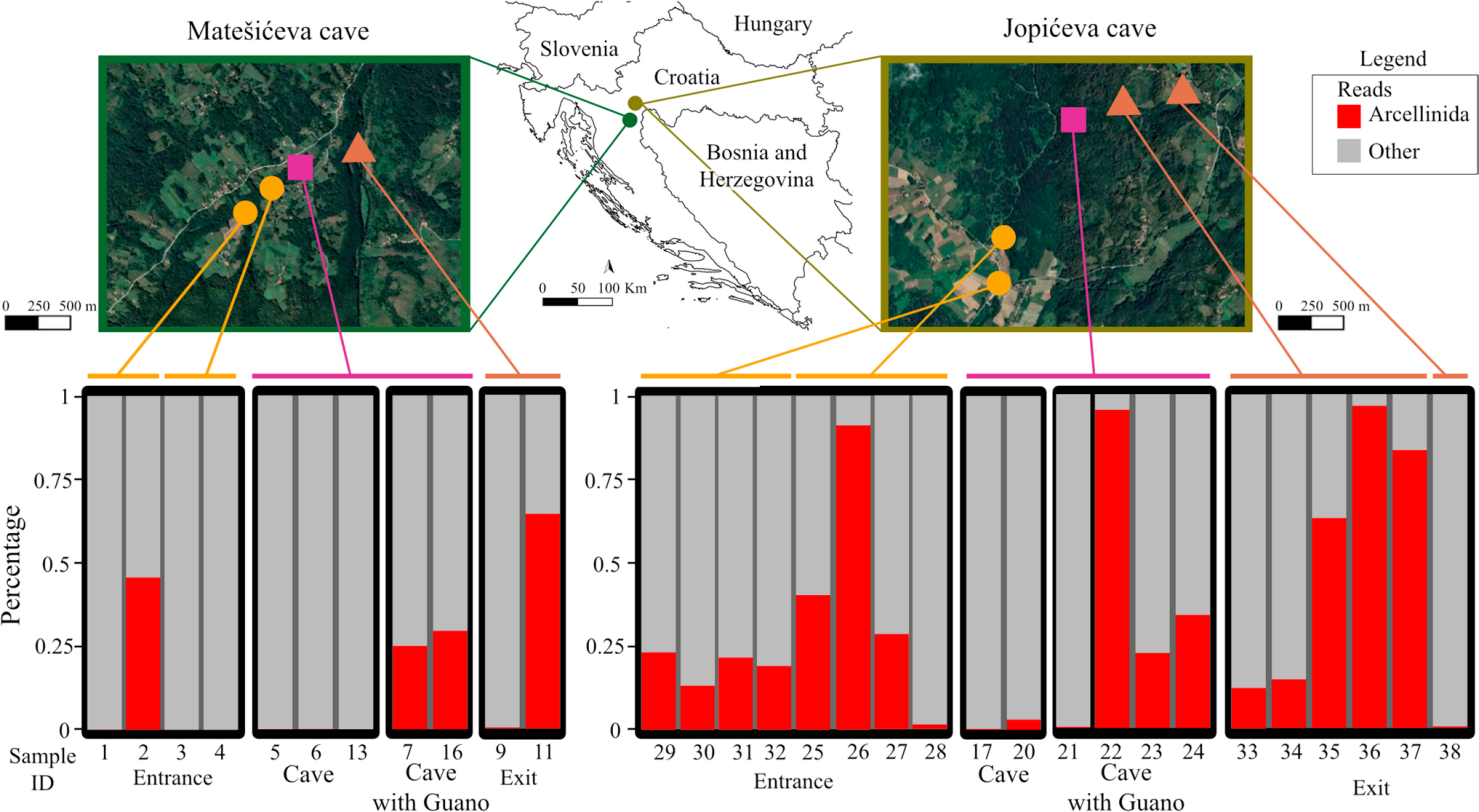
Maps of the localities sampled and percentage of Arcellinida reads per sample as a proxy for abundance

This study was carried out in the Karlovac County of Croatia—more precisely in the Jopićeva cave (cadastral number HR02370, syn. Jopićeva cave-Bent system, 6710 m long) [30] and Matešićeva cave (cadastral number HR00957, syn. Matešićeva-Popovačka cave system, 1246 m long) [31] (Fig. 1). These caves are part of Dinaric karst [32], known as one of the “hotspots of subterranean biodiversity” in the world [33]. Both Jopićeva and Matešićeva cave share similar hydrological characteristics: a surface stream gradually sinks into the karstic terrain and then re-appears within the cave, thus becoming the cave stream. After several hundreds of metres, cave stream exits the cave and forms an exit stream (Fig. 1). Both Matešićeva cave and Jopićeva cave have been designated as Important Underground Sites for Bats in Europe for the genera *Rhinolophus*, *Myotis*, and *Miniopterus* [34]. They support hibernation colonies (Jopićeva cave, ≤ 1545 ind.; Matešićeva cave, ≤ 13 ind.) and maternity colonies (Matešićeva cave, ≤ 690 ind.) and serve as day shelters for bats (Jopićeva cave, ≤ 110 ind.; Matešićeva cave, ≤ 600 ind.) [35]. Guano presence in these caves is variable in terms of quantity, age, spatial and temporal distribution.

In this study, a total of 31 samples, comprising 11 from Matešićeva cave area and 20 from Jopićeva cave area, were collected to investigate the diversity of Arcellinida. The samples were divided into four categories after their characteristics (table S1): (a) entrance stream (sinking stream; surface lotic habitat); (b) cave stream without guano (cave lotic habitat); (c) cave pond with guano (cave lentic habitat); (d) exit stream (formed by water exiting the cave; surface lotic habitats) (Fig. 1).

Aquatic sediments were collected by gently scooping the upper layer of sediment from these habitats using plastic bottles. Immediately after collection, 1 ml of the sampled sediment was transferred into Eppendorf tubes containing a nucleic acid preservation solution (LifeGuard, Qiagen). Subsequently, the contents of the Eppendorf tubes were gently shaken to ensure thorough mixing. Sampling in Matešićeva cave took place on 27 November 2021, while sampling in Jopićeva cave occurred in 5 December of the same year. The water parameter pH and conductivity were measured in all sites in situ using hand instruments (Kestrel 3000, Voltcraft LWT-100, Metrohm 827 pH lab).

### Morphological Observations

Sampled aquatic sediment from caves was examined with a Carl Zeiss Axiostar Plus light microscope for the presence and morphological diversity of Arcellinida using the methodology presented in Baković et al. (2019) [24]. All detected testate amoebae were identified after the following specialized literature [36–38] and original species descriptions [24, 39].

### eDNA Extraction, PCR and Sequencing

Total eDNA was extracted using the DNeasy PowerSoil Pro Kit (Qiagen) following the instructions provided by the manufacturer. We used a semi-nested protocol for amplifying the COI region, as described by González-Miguéns et al. (2023) [29]. In short, we proceeded to an initial amplification with the broad spectrum primers LCO 1490 (5′ GGTCAACAAATCATAAAGATATTGG 3′) and HCO 2198 (5′ TAAACTTCAGGGTGACCAAAAAATCA 3′) [40]. The PCR program included an initial denaturation at 96 °C for 5 min, followed by 40 cycles at 94 °C for 15 s, 40 °C for 15 s, and 72 °C for 90 s, concluding with a final extension step at 72 °C for 10 min. The product from the first PCR, typically diluted at a 1:20 ratio, served as the template for a second amplification using the Arcellinida-specific primer ArCOIR (5′ GGTATTYTAGCWCATTCNRGTGG 3′) [41] paired with LCO. The PCR profile for this stage included an initial denaturation at 96 °C for 5 min, followed by 35 or 40 cycles at 94 °C for 15 s, 55 °C for 15 s, and 72 °C for 90 s, concluding with a final extension step at 72 °C for 10 min. PCR products were quantified using a Qubit 3 fluorometer (Invitrogen), with dsDNA high sensitivity (HS) assay kits (Thermo Fisher). Then, we normalized sample DNA concentration, generating a pool with all the samples. Finally, the pool was sequenced with NovaSeq 500 cycles (Illumina) paired-end 250 bp, by the Genomic Unit of the *Fundación Parque Científico de Madrid*, Spain.

### eDNA Data Curation

Illumina reads were curated following this pipeline based on previous work of González-Miguéns et al*.* (2023) [29]: (1) trimming of primers and demultiplexing was done with Cutadapt ver. 2.8 [42] following the pipeline “Cutadapt_Pipeline.bash”. (2) The resulting reads per sample were analysed with the Dada2 R package [43] following the tutorial in https://benjjneb.github.io/dada2/tutorial_1_8.html, with minor modifications, filtering and trimming after positions 210 and 200 for the forward and reverse, respectively. We dereplicated them, merged the paired reads, generated amplicon sequencing variants (ASVs), and removed the chimeric sequences. (3) For taxonomic assignation, we applied VSEARCH ver. 2.14 [44] using a curated database based from González-Miguéns et al*.* (2024) [45]. Each ASV with a percentage of identity value higher than 82% with known Arcellinida sequences compared with the GenBank database to confirm its assignation to Arcellinida.

### ASVs Clustering into OTUs and Phylogenetic Analysis

The ASVs that were assigned to Arcellinida were aligned with the Arcellinida COI sequence data of González-Miguéns et al*.* (2022) [46] and the metabarcoding data of González-Miguéns et al. (2024) [45] using the MAFFT auto algorithm [47] as implemented in Geneious version 2019.0.4. Next, we proceeded to tree building; tree topologies and node supports were evaluated with maximum likelihood (ML), using IQTREE2 ver. 2.0 [48]. The best substitution models were selected with ModelFinder [49], implemented in IQ-TREE2 ver. 2.0. Node supports were assessed with 5000 ultrafast bootstrap replicate approximation [50, 51].We used the R package ggtree ver. 3.2 [52] and its related packages to graphically represent the tree with ecological and taxonomic information related to each ASV. Finally, we generated a distance matrix to cluster the ASVs in operational taxonomic units (OTUs) using the R package DECIPHER ver. 2.22 [53]. In that purpose, we generated two OTUs datasets (to test if how far the “(meta)barcoding gap”, the genetic breadth of the study unit influenced our inferences), one clustering the ASVs using a pairwise distance of 3% and a second of 4% as a threshold.

### Diversity Metrics

We plotted the total number of ASVs, OTUs and percentage of Arcellinida reads, with respect to the total read numbers per sample and the total number of these variables with respect of the total reads of each sample using ggplot2 [54]. Then, we drew Venn diagrams to visualize how far ASVs and OTUs were shared by the different ecosystem types. Furthermore, we generate hierarchical cluster, by distance, that represents differences among types of sampling sites in terms of biotic composition (presence and absence of OTUs) and abiotic factors (pH and conductivity). Finally, we illustrated haplotype networks for the OTUs shared between at least two ecosystem types, to infer the “intraspecific” patterns. For that purpose, we used PopART software ver. 1.7 [55], using a TCS network.

## Results

The number of Illumina reads per sample through dada2 pipeline is detailed in Table S2. The percentage of ASVs assigned to Arcellinida was lower within caves without guano than outside (average of cave without guano 0.74%, cave with guano 34.4%, entrance and exit of the cave 23.68 and 41.81%, respectively) (Fig. 1 and Table S3). Among subterranean habitats, sites enriched with bat guano hosted a larger percentage of Arcellinida sequences (83.6%) (Fig. 1 and Table S3).

**Fig. 2:**
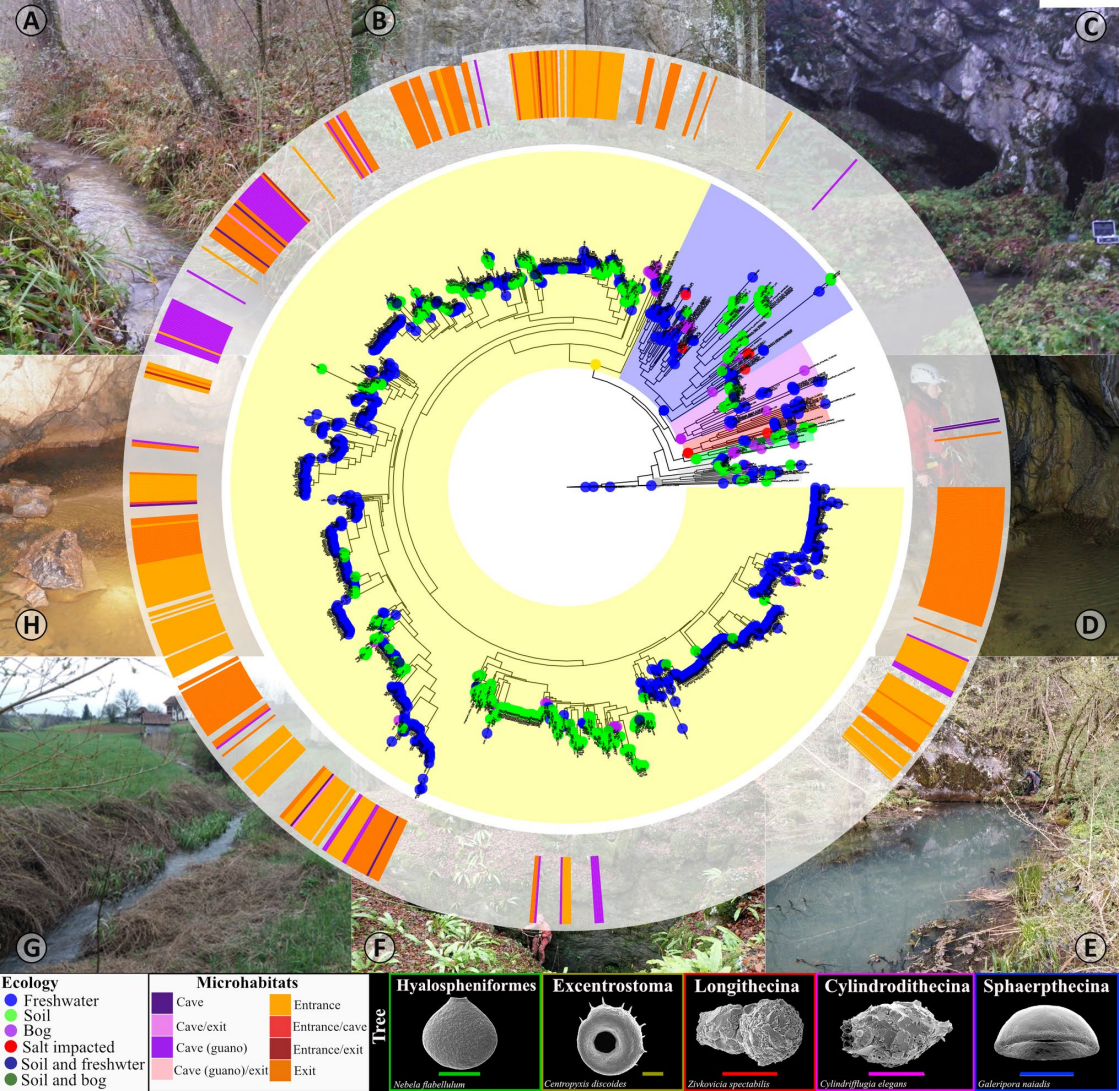
Maximum likelihood phylogenetic tree of Arcellinida based on mitochondrial cytochrome oxidase subunit I (COI). Colour dots at the tips of branches represent the ecology of organisms. Background colours represent infraorders in Glutinoconcha. The outer circle represents the microhabitats type from which sequences are derived; it was specified with a different colour when the same ASV was found in two different microhabitats. Pictures represent the localities where the samples were taken: **A** Entrance stream in Matešićeva cave site, **B** entrances to the Matešićeva cave, **C** exit stream from the Matešićeva cave, **D** cave pond with bats guano in Jopićeva cave, **E** exit stream from Jopićeva cave, **F** Jopićeva cave entrance, **G** entrance stream in Jopićeva cave site, **H** cave stream in Matešićeva cave

We found a total of 485 Arcellinida ASVs in this study (Fig. 2). The phylogenetic tree based on ASVs with the rest of the sequences revealed that the majority (94.42%) belonged to the infraorder Excentrostoma (including for instance genera *Centropyxis* and *Plagiopyxis*) (Fig. 2). A few ASVs (4.99%) were others affiliated to Sphaerothecina (*Netzelia, Arcella*) and the suborder Phryganellina (*Cryptodifflugia*). These molecular data align with morphological observations, which primarily identified species from the suborder Excentrostoma (seven species) (Table S4), particularly those belonging to the genus *Centropyxis*, such as *C. aerophila*. Moreover, we also found species recently described exclusively in the Dinaric karst caves and an isolated karst area in Croatia, like *C. bipilata* [24, 39]. Additionally, species morphologically assigned to the genus *Difflugia* (one species), corresponding to the suborder Glutinoconcha [46], and *Cryptodifflugia* (two species), within the suborder Phryganellina [56], have also been found. Of the total of ASVs, six of them had already been detected in a previous study [45], four in Switzerland and two in Spain; all these sequences were found here outside the cave system; in other regions, these sequences were found in moss, soil or freshwater sediments. We almost did not observe any differences in biodiversity assessment when using the two different thresholds for OTU separation (3% vs. 4%). The difference between the number of OTUs obtained with a threshold of 3% and 4% was 0 in almost all samples, the samples had the same number of OTUs with thresholds of 3% and 4%, except between samples 34 (threshold 3% = 4 Arcellinida OTUs and threshold 4% = 3) and 36 (threshold 3% = 22 Arcellinida OTUs and threshold 4% = 19) (Table S3). Therefore, we used the OTU dataset generated with pairwise distances of 4%. As it was the most restrictive approach, we hoped to increase the robustness of our inferences.

**Fig. 3:**
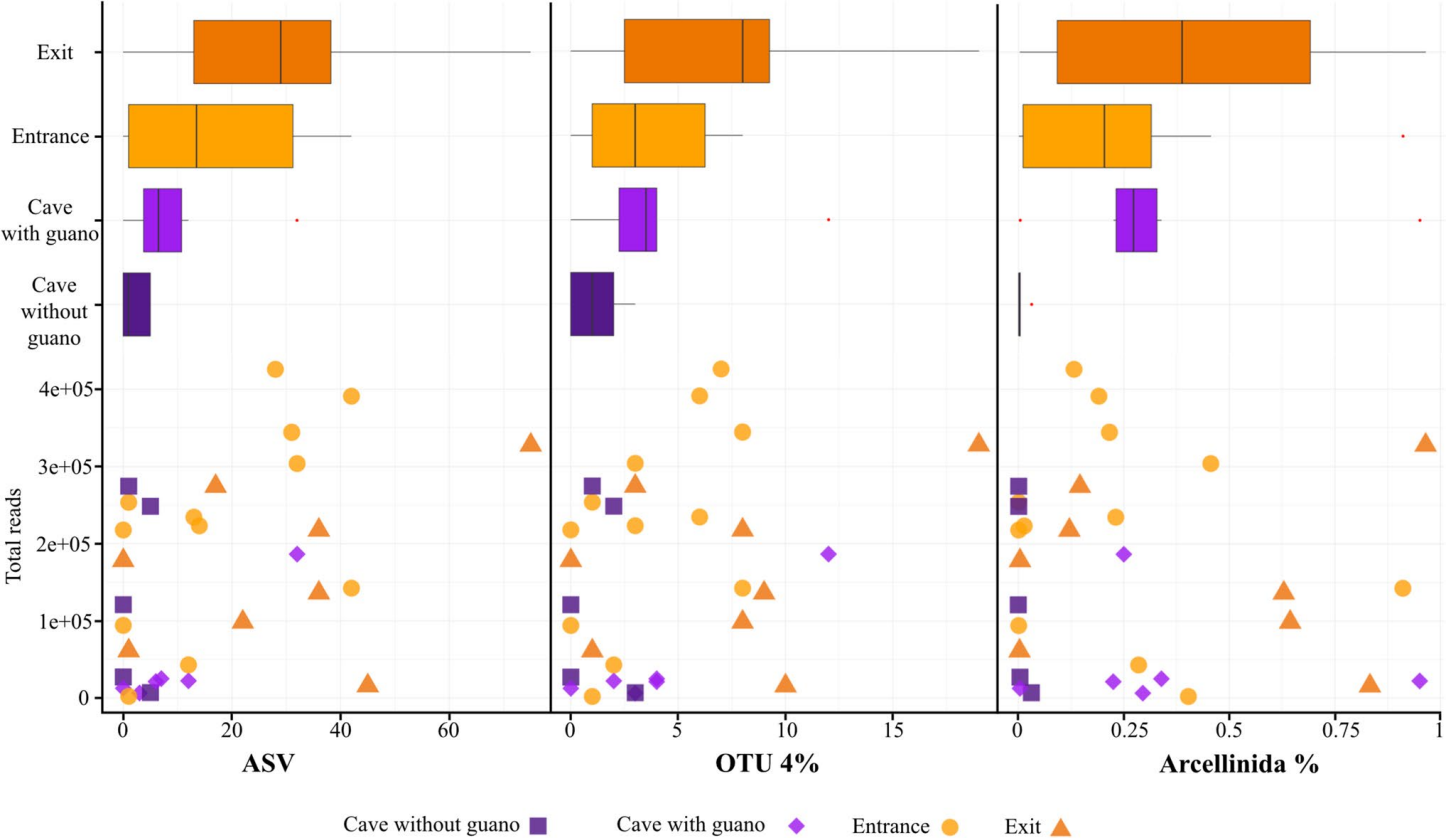
Plot representing read numbers (Y axis) *vs.* ASVs (amplicon sequence variants), OTUs (operational taxonomic units) numbers and the percentage of Arcellinida in each sample (each point corresponds to a site; X axis). The boxplots represents the values of the X axis in each case (ASVs, OTUs and percentage of Arcellinida reads)

**Fig. 4:**
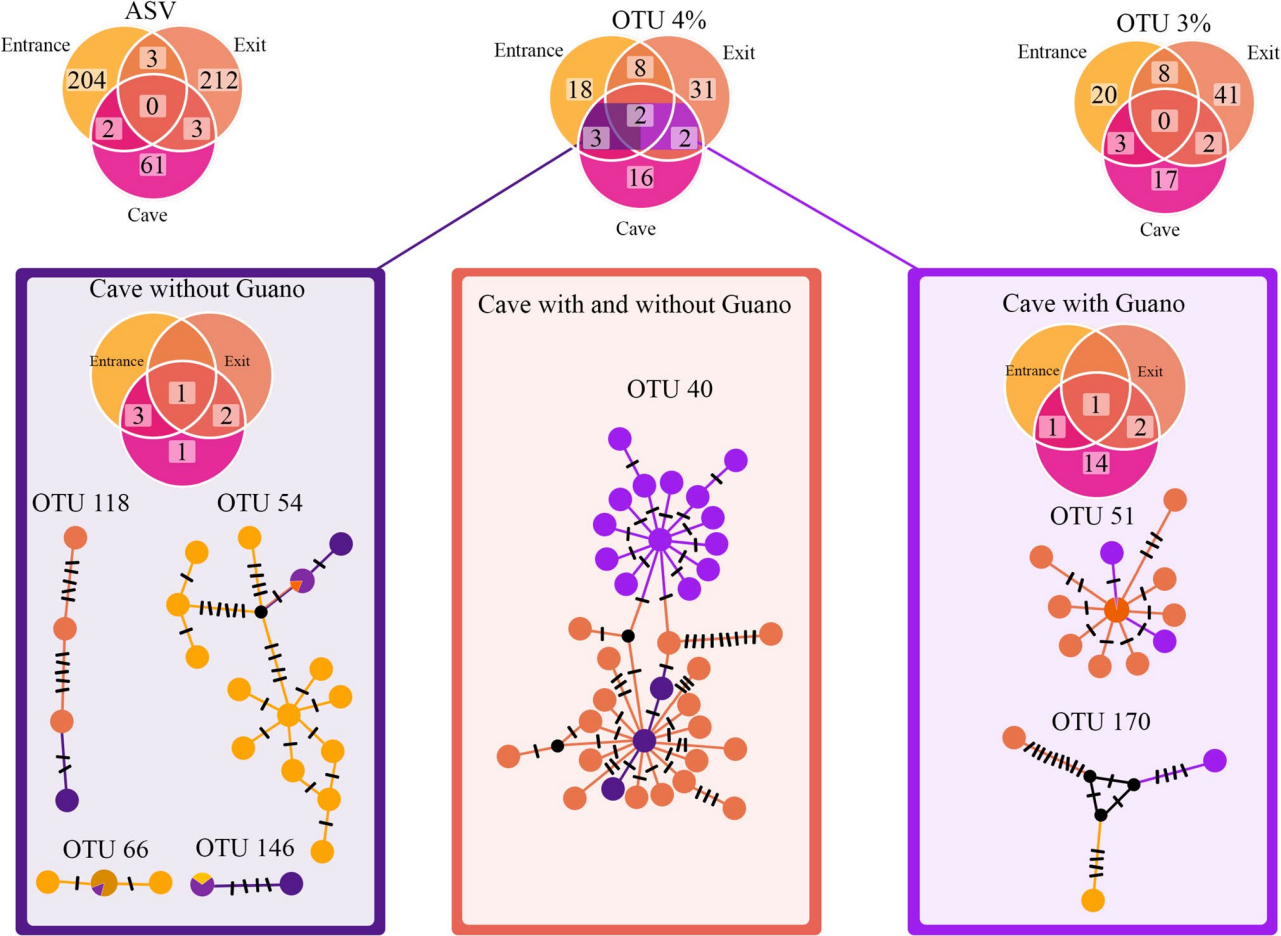
Venn diagrams representing the distribution of diversity, as represented by ASVs, OTUs (4%) and OTUs (3%). Some illustrative examples of haplotype networks have been built for cave OTUs shared with outside. Light purple represent ASVs from guano-sites, dark purple represents ASVs from sites in caves without guano, light orange represents ASVs from entrance-sites and dark orange represents ASVs from exit-sites

Arcellinida communities were more diverse outside of the cave than inside, in terms of both ASV and OTUs and percentage of Arcellinida reads (Fig. 3). However, community composition associated with guano differed deeply from those of other cave environments. Firstly, they presented low levels of connectivity with any other surveyed habitat (Figs. 2 and 4). Indeed, among all OTUs found in the cave without guano, only one was not detected outside (Fig. 4). On the other hand, almost all OTUs found in guano impacted sites were not found elsewhere. At the infraspecific level (ASVs), OTUs found outside guano-impacted sites did not present any haplotypic structure, while most of the ASVs diversity came from the outside (cave entrance and exit). The two OTUs that were shared between guano and not guano cave environments (OTU 40 and 170) present a clear haplotypic structure corresponding to each microhabitat type.

**Fig. 5:**
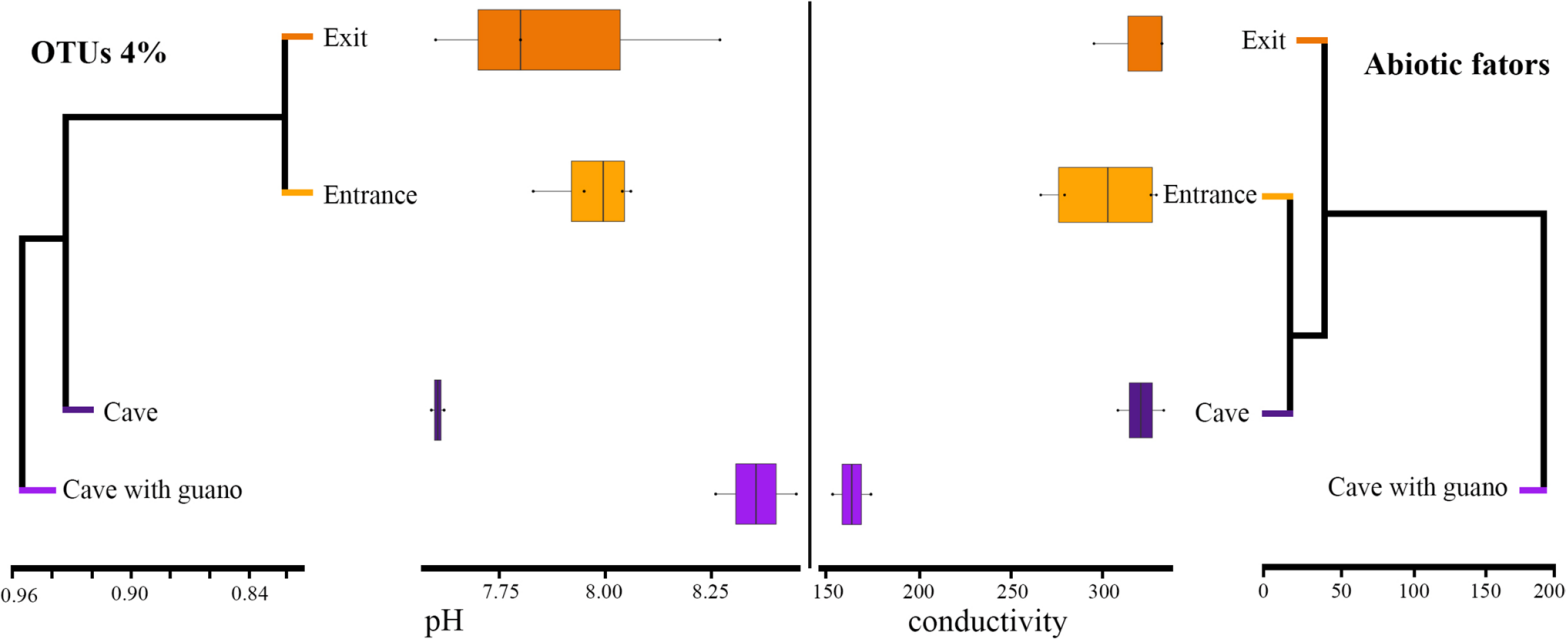
A comparison of hierarchical clustering by distance of sample types, representing Arcellinida community composition (left) versus abiotic factors (right)

In support for a separation of the guano-associated communities from the rest, a clustering analysis evidenced their specificity. This is in line with abiotic factors (pH and conductivity) which appear different from the other microhabitats (Fig. 5).

## Discussion

### Abundance and Diversity of Arcellinida in Caves

This study shows that without any influence of the guano, Arcellinida communities are poorer inside than outside of the caves. This is reflected in both ASVs and OTUs numbers, which are in average lower (Figs. 1 and 3, Table S3). Moreover, Arcellinida sequence reads have higher relative abundance in guano impacted sites than anywhere else (Fig. 1). While metabarcoding results can be considered at best semi-quantitative for different reasons [57], it has been shown that high ratios of Arcellinida sequences versus other sequences can be correlated with higher population densities [45]. Our estimations based on Arcellinida read abundance are therefore in line with the number of species that we observed with microscopy (Table S4). Besides, sampling sites within and outside the cave share similar abiotic values (pH and conductivity) (Fig. 5), and communities composition within the cave can be considered, to a large extent, as a subset of those from the outside (Fig. 4). Communities at the cave entrance share most OTUs with the exit, which suggests a good interconnection between all these sampling sites. The lack of nutrients within the cave is probably responsible for the low densities of Arcellinida, as inferred by low proportions of Arcellinida reads found within the caves. The low infraspecific diversity (i.e. low ASV/OTU ratios Table S3) has been shown to indicate unstable Arcellinida populations, as diversity might be regularly wiped out by floods [45]. This might suggest that there are no stable Arcellinid communities within the cave without guano, which would starve by lack of nutrients; caves would act as ecological traps for individuals brought in by the river current. This situation changes drastically when nutrients (such as guano) are present in sufficient amounts, promoting a diversity of guano-associated communities.

### Diversity of Guano-Associated Communities

Guano emerges as a pivotal energy source within cave food webs [58, 59] contributing with organic matter to an oligotrophic system [4, 60], which is necessary for supporting heterotrophic eukaryotes growth. Those environments shaped by bat guano create favourable conditions for the development of several yeast species as well [61] as well as protists [5, 62]. These microorganisms are typical preys for Arcellinida and could sustain their populations. In line, we observed significantly higher proportions of Arcellinida reads in guano-influenced cave samples than in cave stream samples without guano; these proportions were even comparable to epigeous samples (Figs. 1 and 3).

Guano-impacted sites did not only have more abundant Arcellinida populations than sites without guano, but they were also more diverse in the number of ASV and OTUs (Fig. 4). Observational studies by Baković et al*.* [39, 63] identified the highest richness in taxonomic groups associated with this microhabitat. Other studies found that, in heterogeneous cave environments that include guano-impacted sites, testate amoeba species diversity was similar to epigeous communities such as those that inhabit soil litter or mosses [23]. High ASV/OTU values indicated high infraspecific diversity, which suggests that Arcellinida form stable populations in guano-impacted sites (Fig. 4). The reduced amount of OTUs in common between guano and non-guano communities suggests the existence of a specific diversity associated with bat droppings, in other terms, “guanophilic” Arcellinida communities. These observations are in accordance with previous studies conducted on cave fauna. For instance, communities of arthropods associated with bat guano are apparently more dependent on the guano microenvironment itself than on the overall cave environment [59]. In general, guanophilous species often dominate cave faunas [64].

How far guano-associated Arcellinida are truly specific from Dinaric Karst caves still remains to be determined. While we detected six “non-guano” ASVs that were also found in previous studies dealing with surface samples in Switzerland and in Spain, respectively [29, 45], no guano-associated ASVs were found in any other locality. Future Arcellinida metabarcoding studies will reveal how far the species detected here were truly restricted to bat colonies in the Dinaric Karst. Taking example in animals, troglobiotic species are frequent [65–68], and it seems therefore likely that Arcellinida follow the same pattern. In these environments, the Arcellinida, which belong mostly to genera *Centropyxis* and *Plagiopyxis* (Fig. 2), would act as top predators of the subterranean microbiota, by controlling decomposers and other micro predators which process the guano released by bats in the Dinaric karst, like they would do in other ecosystems [69–71]. They can be expected, therefore, to be key elements in the ecological equilibria of karstic ecosystems.

## Conclusions

This study highlights the contrasting communities of Arcellinida within caves, which seem to depend on guano as a critical energy source. In the absence of guano, cave Arcellinida populations exhibit lower diversity and abundance compared to external environments, likely due to nutrient scarcity and unstable conditions that prevent the establishment of stable communities. Caves without guano host communities that are subsets of external populations, most likely brought in by river currents. Conversely, guano-enriched sites foster higher Arcellinida diversity and abundance, supported by the nutrient-rich guano input. These “guanophilic” communities appear distinct and may include species potentially endemic to the Dinaric Karst. The role of Arcellinida as top predators in guano-impacted microbiota underscores their ecological significance, controlling decomposers and other micro-predators within these unique karstic ecosystems. Future studies are necessary to elucidate the specificity and broader ecological roles of guano-associated Arcellinida in these environments.

## Supplementary Information

Below is the link to the electronic supplementary material.

Supplementary file1 (DOCX 49 KB)

## Data Availability

The raw data sequences generated in this study can be download from SRA NCBI, under the accession code PRJNA1139688.
