# Supplementary material for: Predators in the Dark: Metabarcoding Reveals Arcellinida Communities Associated with Bat Guano, Endemic to Dinaric Karst in Croatia
Source: Microb Ecol. 2025 Jan 6;87(1):166. doi: 10.1007/s00248-024-02483-z (PMC11703892; doi:10.1007/s00248-024-02483-z)
Supplement: Supplementary file 1 — Supplementary file1 (DOCX 49 KB) [file 248_2024_2483_MOESM1_ESM.docx]

Supplemental Information for:

Predators in the Dark: Metabarcoding reveals Arcellinida communities associated with bat guano, endemic to Dinaric Karst in Croatia

Ángel García-Bodelón^1,2 *^, Najla Baković^3,4^, Emilio Cano^1, 5^, Fernando Useros^1^, Enrique Lara^1^, Rubén González-Miguéns^1,6, *^

^1^ Real Jardín Botánico (RJB-CSIC), C/ Moyano 1, 28014 Madrid, Spain

^2^ Universidad Complutense de Madrid, 28040, Madrid, Spain

^3^ Laboratory for flora, fauna and habitats, DVOKUT-ECRO Ltd, Croatia

^4^ADIPA – Society for Research and Conservation of Croatian Natural Diversity, Croatia

^5^ Research Support Unit, Real Jardín Botánico (CSIC), C/ Moyano 1, 28014 Madrid, Spain

^6^ Institut de Biologia Evolutiva (CSIC-Universitat Pompeu Fabra), 08003, Barcelona, Spain

| Supplementary tables | |
| --- | --- |
| Table S1 | Page 2 |
| Table S2 | Page 3 |
| Table S3 | Page 4 |
| Table S4 | Page 5 |

Table S1. Ecology, pH, conductivity and collection date from caves per sample.

| Id. | Ecology | pH | Conductitivity  (µS/cm) | Date | Latitude and longitude |
| --- | --- | --- | --- | --- | --- |
| **Matešićeva cave** | | | | | |
| Entrance | | | | | |
| Sample1 | Freshwater sediment | 7.95 | 266 | 27-Nov-2021 | 45.11147, 15.61127 |
| Sample2 | Freshwater sediment | 7.95 | 266 | 27-Nov-2021 | 45.11147, 15.61127 |
| Sample3 | Freshwater sediment | 7.83 | 279 | 27-Nov-2021 | 45.11147, 15.61127 |
| Sample4 | Freshwater sediment | 7.83 | 279 | 27-Nov-2021 | 45.11147, 15.61127 |
| Cave without guano | | | | | |
| Sample5 | Freshwater sediment | 7.59 | 308 | 27-Nov-2021 | 45.11147, 15.61127 |
| Sample6 | Freshwater sediment | 7.59 | 308 | 27-Nov-2021 | 45.11147, 15.61127 |
| Sample13 | Freshwater sediment | 7.59 | 308 | 27-Nov-2021 | 45.11147, 15.61127 |
| Cave with guano | | | | | |
| Sample7 | Freshwater sediment | 8.26 | 152 | 27-Nov-2021 | 45.11147, 15.61127 |
| Sample16 | Freshwater sediment | 8.26 | 152 | 27-Nov-2021 | 45.11147, 15.61127 |
| Exit | | | | | |
| Sample9 | Freshwater sediment | 8.27 | 295 | 27-Nov-2021 | 45.11147, 15.61127 |
| Sample11 | Freshwater sediment | 8.27 | 295 | 27-Nov-2021 | 45.11147, 15.61127 |
| **Jopićeva cave** | | | | | |
| Entrance | | | | | |
| Sample25 | Freshwater sediment | 8.06 | 326 | 05-Dec-2021 | 45.29505, 15.58700 |
| Sample26 | Freshwater sediment | 8.06 | 326 | 05-Dec-2021 | 45.29505, 15.58700 |
| Sample27 | Freshwater sediment | 8.06 | 326 | 05-Dec-2021 | 45.29505, 15.58700 |
| Sample28 | Freshwater sediment | 8.06 | 326 | 05-Dec-2021 | 45.29505, 15.58700 |
| Sample29 | Freshwater sediment | 8.04 | 329 | 05-Dec-2021 | 45.29505, 15.58700 |
| Sample30 | Freshwater sediment | 8.04 | 329 | 05-Dec-2021 | 45.29505, 15.58700 |
| Sample31 | Freshwater sediment | 8.04 | 329 | 05-Dec-2021 | 45.29505, 15.58700 |
| Sample32 | Freshwater sediment | 8.04 | 329 | 05-Dec-2021 | 45.29505, 15.58700 |
| Cave without guano | | | | | |
| Sample17 | Freshwater sediment | 7.62 | 333 | 05-Dec-2021 | 45.29505, 15.58700 |
| Sample20 | Freshwater sediment | 7.62 | 333 | 05-Dec-2021 | 45.29505, 15.58700 |
| Cave with guano | | | | | |
| Sample21 | Freshwater sediment | 8.45 | 171 | 05-Dec-2021 | 45.29505, 15.58700 |
| Sample22 | Freshwater sediment | 8.45 | 171 | 05-Dec-2021 | 45.29505, 15.58700 |
| Sample23 | Freshwater sediment | 8.45 | 171 | 05-Dec-2021 | 45.29505, 15.58700 |
| Sample24 | Freshwater sediment | 8.45 | 171 | 05-Dec-2021 | 45.29505, 15.58700 |
| Exit | | | | | |
| Sample33 | Freshwater sediment | 7.6 | 332 | 05-Dec-2021 | 45.29505, 15.58700 |
| Sample34 | Freshwater sediment | 7.6 | 332 | 05-Dec-2021 | 45.29505, 15.58700 |
| Sample35 | Freshwater sediment | 7.6 | 332 | 05-Dec-2021 | 45.29505, 15.58700 |
| Sample36 | Freshwater sediment | 7.6 | 332 | 05-Dec-2021 | 45.29505, 15.58700 |
| Sample37 | Freshwater sediment | 7.8 | 332 | 05-Dec-2021 | 45.29505, 15.58700 |
| Sample38 | Freshwater sediment | 7.8 | 332 | 05-Dec-2021 | 45.29505, 15.58700 |

Table S2. Number of Illumina reads per sample through the DADA2 pipeline.

|  | input | filtered | denoisedF | denoisedR | merged | nonchim | percentage |
| --- | --- | --- | --- | --- | --- | --- | --- |
| Sample1 | 143955 | 109396 | 108559 | 108412 | 98595 | 94107 | 65.37 |
| Sample2 | 431820 | 360226 | 358766 | 359038 | 341674 | 304093 | 70.42 |
| Sample3 | 324523 | 270265 | 269184 | 269580 | 256495 | 217907 | 67.15 |
| Sample4 | 353675 | 294882 | 293191 | 293741 | 281349 | 253753 | 71.75 |
| Sample5 | 336816 | 281079 | 279519 | 279381 | 272752 | 248615 | 73.81 |
| Sample6 | 416118 | 332924 | 331609 | 330746 | 289354 | 274709 | 66.02 |
| Sample7 | 284903 | 237525 | 235587 | 235896 | 209481 | 186547 | 65.48 |
| Sample9 | 89003 | 67719 | 66748 | 66412 | 62551 | 61277 | 68.85 |
| Sample11 | 147570 | 118374 | 117403 | 117242 | 107771 | 98529 | 66.77 |
| Sample13 | 166826 | 134703 | 134367 | 134120 | 125413 | 121011 | 72.54 |
| Sample16 | 10515 | 8270 | 7968 | 7940 | 6892 | 6445 | 61.29 |
| Sample17 | 34947 | 29618 | 29391 | 29141 | 27568 | 27218 | 77.88 |
| Sample20 | 9134 | 7672 | 7500 | 7501 | 7326 | 7021 | 76.87 |
| Sample21 | 17459 | 14245 | 13931 | 13822 | 13276 | 12657 | 72.5 |
| Sample22 | 27205 | 23447 | 23077 | 23235 | 22632 | 22262 | 81.83 |
| Sample23 | 31282 | 25037 | 24173 | 24056 | 21639 | 21307 | 68.11 |
| Sample24 | 41349 | 33308 | 32115 | 32130 | 26580 | 25084 | 60.66 |
| Sample25 | 4731 | 3389 | 2785 | 2703 | 2229 | 2151 | 45.47 |
| Sample26 | 193874 | 162703 | 161923 | 161683 | 154875 | 142426 | 73.46 |
| Sample27 | 60044 | 49121 | 48614 | 48609 | 47572 | 43004 | 71.62 |
| Sample28 | 274435 | 229619 | 229100 | 228274 | 224112 | 223271 | 81.36 |
| Sample29 | 315256 | 264633 | 263045 | 262849 | 256057 | 234552 | 74.4 |
| Sample30 | 576143 | 486125 | 482889 | 482675 | 458780 | 425803 | 73.91 |
| Sample31 | 459146 | 388011 | 385780 | 385218 | 368973 | 344372 | 75 |
| Sample32 | 587860 | 491228 | 486487 | 485966 | 463621 | 390963 | 66.51 |
| Sample33 | 300738 | 248316 | 246151 | 245872 | 237750 | 217784 | 72.42 |
| Sample34 | 363200 | 301788 | 300445 | 300414 | 290741 | 274566 | 75.6 |
| Sample35 | 227827 | 176903 | 175289 | 174727 | 168666 | 136232 | 59.8 |
| Sample36 | 440839 | 368147 | 366206 | 366126 | 350681 | 328016 | 74.41 |
| Sample37 | 207317 | 175180 | 173970 | 173908 | 169114 | 159280 | 76.83 |
| Sample38 | 237071 | 194824 | 193026 | 193510 | 182453 | 178273 | 75.2 |

Table S3. Number of ASVs, OTUs with 3% threshold, OTUs with 4% threshold, ASV/OUT, total reads and percentage of Arcellinida per sample.

|  | ASV | OTU 3% | OTU 4% | ASV/OUT 4% | Total reads | % Arcellinida |
| --- | --- | --- | --- | --- | --- | --- |
| Sample1 | 0 | 0 | 0 | 0 | 94,107 | 0.001 |
| Sample2 | 32 | 3 | 3 | 10.666 | 304,093 | 0.456 |
| Sample3 | 0 | 0 | 0 | 0 | 217,907 | 0.001 |
| Sample4 | 1 | 1 | 1 | 1 | 253,753 | 0.001 |
| Sample5 | 5 | 2 | 2 | 2.5 | 248,615 | 0.001 |
| Sample6 | 1 | 1 | 1 | 1 | 274,709 | 0.001 |
| Sample7 | 32 | 12 | 12 | 2.666 | 186,547 | 0.250 |
| Sample9 | 1 | 1 | 1 | 1 | 61,277 | 0.003 |
| Sample11 | 22 | 8 | 8 | 2.75 | 98,529 | 0.644 |
| Sample13 | 0 | 0 | 0 | 0 | 121,011 | 0 |
| Sample16 | 3 | 3 | 3 | 1 | 6,445 | 0.295 |
| Sample17 | 0 | 0 | 0 | 0 | 27,218 | 0.004 |
| Sample20 | 5 | 3 | 3 | 1.666 | 7,021 | 0.031 |
| Sample21 | 0 | 0 | 0 | 0 | 12,657 | 0.004 |
| Sample22 | 12 | 2 | 2 | 6 | 22,262 | 0.951 |
| Sample23 | 6 | 4 | 4 | 1.5 | 21,307 | 0.225 |
| Sample24 | 7 | 4 | 4 | 1.75 | 25,084 | 0.339 |
| Sample25 | 1 | 1 | 1 | 1 | 2,151 | 0.403 |
| Sample26 | 42 | 8 | 8 | 5.25 | 142,426 | 0.911 |
| Sample27 | 12 | 2 | 2 | 6 | 43,004 | 0.285 |
| Sample28 | 14 | 3 | 3 | 4.666 | 223,271 | 0.014 |
| Sample29 | 13 | 6 | 6 | 2.166 | 234,552 | 0.231 |
| Sample30 | 28 | 7 | 7 | 4 | 425,803 | 0.132 |
| Sample31 | 31 | 8 | 8 | 3.875 | 344,372 | 0.216 |
| Sample32 | 42 | 6 | 6 | 7 | 390,963 | 0.191 |
| Sample33 | 36 | 8 | 8 | 4.5 | 217,784 | 0.121 |
| Sample34 | 17 | 4 | 3 | 4.25 | 274,566 | 0.146 |
| Sample35 | 36 | 9 | 9 | 4 | 136,232 | 0.629 |
| Sample36 | 75 | 22 | 19 | 3.947 | 328,016 | 0.965 |
| Sample37 | 45 | 10 | 10 | 4.5 | 159,280 | 0.833 |
| Sample38 | 0 | 0 | 0 | 0 | 17,8273 | 0.004 |

Table S4. List of the morphotypes identified in the Matešićeva cave and Jopićeva cave.

| **Matešićeva cave** | | **Jopićeva cave** | |
| --- | --- | --- | --- |
| **cave stream** | **cave pond with guano** | **cave stream** | **cave pond with guano** |
| *Centropyxis aculeata* | *Centropyxis aerophila* | *Centropyxis plagiostoma* | *Centropyxis aerophila* |
| *Centropyxis aerophila* | *Centropyxis bipilata** | cf. *Difflugia* (22.5 μm) | *Centropyxis bipilata** |
| cf. *Difflugia* (15 μm) | cf. *Difflugia* (17.5 μm) | cf. *Difflugia* (42.5 μm) | cf. *Difflugia* (47.5 μm) |
| cf. *Difflugia* (27.5 μm) | cf. *Difflugia* (35 μm) | cf. *Difflugia* (60 μm) | cf. *Difflugia* sp*.* (22.5 μm) |
| *Psammonobiotus dinarica** | *Cryptodifflugia oviformis* | cf. *Pyxidicula/Parmulina* | Unidentified testate amoebae** |
| *Cryptodifflugia oviformis* | *Microchlamys patella* | *Psammonobiotus dinarica** | *Cyclopyxis eurystoma* |
| *Microchlamys patella* | *Plagiopyxis declivis* | *Cryptodifflugia sp.* |  |
|  | Unidentified testate amoebae (42.5 μm) | *Difflugia* cf*. oblonga* |  |
|  |  | *Microchlamys patella* |  |

*Recently described species found exclusively in the Dinaric karst caves and one isolated karst area in Croatia (Baković et al., 2023, 2019)

** Candidate for new species for science
